# Semaglutide Treatment Effects on Liver Fat Content in Obese Subjects with Metabolic-Associated Steatotic Liver Disease (MASLD)

**DOI:** 10.3390/jcm13206100

**Published:** 2024-10-13

**Authors:** Tereza Dusilová, Jan Kovář, Ivana Laňková, Lenka Thieme, Monika Hubáčková, Petr Šedivý, Dita Pajuelo, Martin Burian, Monika Dezortová, Denisa Miklánková, Hana Malínská, Petra Svobodová Šťastná, Rudolf Poledne, Milan Hájek, Martin Haluzík

**Affiliations:** 1 Institute for Clinical and Experimental Medicine, 140 21 Prague, Czech Republic; tereza.dusilova@ikem.cz (T.D.); ivana.lankova@ikem.cz (I.L.); lenka.thieme@ikem.cz (L.T.); monika.hubackova@ikem.cz (M.H.); petr.sedivy@ikem.cz (P.Š.); dita.pajuelo@ikem.cz (D.P.); martin.burian@ikem.cz (M.B.); monika.dezortova@ikem.cz (M.D.); denisa.miklankova@ikem.cz (D.M.); hana.malinska@ikem.cz (H.M.); petra.stastna@ikem.cz (P.S.Š.); rudolf.poledne@ikem.cz (R.P.); milan.hajek@ikem.cz (M.H.); martin.haluzik@ikem.cz (M.H.); 2 Department of Physiology, Faculty of Science, Charles University, 128 44 Prague, Czech Republic

**Keywords:** obesity, metabolic-dysfunction associated steatotic liver disease, nonalcoholic fatty liver disease, GLP-1 receptor agonists, semaglutide, liver fat, insulin resistance, de novo lipogenesis

## Abstract

**Background**: Metabolic-dysfunction-associated steatotic liver disease (MASLD) represents a major clinical complication of obesity. **Methods**: In this study, we used magnetic resonance (MR) methods to determine the effect of obesity treatment with semaglutide, a GLP-1 receptor agonist, on the liver fat content and selected metabolic variables. We investigated whether treatment would affect the acute response of liver fat to glucose and fructose administration and whether it would affect the fatty acid profile of VLDL-triglycerides. Sixteen obese non-diabetic men underwent a 16-week dietary intervention and 16-week treatment with subcutaneous semaglutide in a crossover design without a washout period. The order of the interventions was randomized. **Results**: After treatment, body weight of the subjects decreased by 5% and liver fat by a third, whereas dietary intervention had no impact on these parameters. The decrease in liver fat with semaglutide did not correlate with changes in body weight and other measures of adiposity and was unrelated to improved insulin sensitivity. **Conclusions**: The proportion of palmitic and palmitoleic acids in VLDL-triglycerides decreased after treatment, suggesting that the beneficial effects of semaglutide on liver fat are mediated by the suppression of de novo lipogenesis.

## 1. Introduction

Obesity and its chronic complications are among the biggest challenges of the healthcare systems around the world. Metabolic-dysfunction-associated steatotic liver disease (MASLD), formerly referred to as non-alcoholic fatty liver disease (NAFLD), is one of the major clinical complications of obesity. It affects 30% of the world’s adult population [1,2] and approximately 75% of patients with obesity [3]. The disease can progress from simple steatosis to steatohepatitis (MASH) and, in some subjects, even to cirrhosis and hepatocellular carcinoma; currently, MASLD is becoming the leading indication for liver transplantation [4].

Lifestyle and dietary interventions are not very effective in the treatment of obesity, and new drugs are still being sought to help patients reduce body weight. Among these drugs, glucagon-like peptide 1 (GLP-1) agonists are among the most promising classes. These drugs, originally intended for the treatment of diabetes due to their incretin action, effectively reduce body weight and liver fat content [5,6,7]. Such an effect of GLP-1 agonists on hepatic fat is usually explained by a decrease in total body fat and a beneficial effect on hepatic insulin resistance [8], but the exact mechanism of action has not yet been characterized.

In our studies of nutrient-induced acute changes in hepatic fat content (HFC), we have shown that repeated glucose administration (3 × 50 g at 2 h intervals) induces a 15% decrease in HFC in nonobese healthy subjects [9]; no such effect was observed in nonobese subjects with liver steatosis [10]. This suggests that in healthy subjects, de novo lipogenesis could not compensate for the loss of hepatic triglycerides by oxidation and secretion in VLDL. It is not quite clear whether the decrease in HFC due to GLP-1 agonist treatment can be associated with an improved response of liver fat to glucose administration.

It has been demonstrated that de novo lipogenesis is a critical contributor to increased hepatic fat in MASLD patients [11], and another mechanism that can explain the effects of GLP-1 receptor agonists on liver fat is reduced de novo lipogenesis.

In this study, we therefore sought to determine whether treatment with the GLP-1 receptor agonist semaglutide (Ozempic^®^) results in a reduction in HFC and by what mechanism such an effect is achieved. We therefore addressed the question of whether semaglutide improves the acute HFC response to repeated loads of glucose and fructose. To determine whether semaglutide treatment affects de novo lipogenesis, we analyzed the fatty acid profile of very-low-density lipoprotein-triglycerides (VLDL-TG). This study was carried out on obese subjects with abdominal obesity who had already developed MASLD.

Overall, our study aimed to contribute to the understanding of the mechanism of reduction in liver fat after semaglutide treatment.

## 2. Materials and Methods

### 2.1. Subjects and Design of the Study

**Figure 1:**
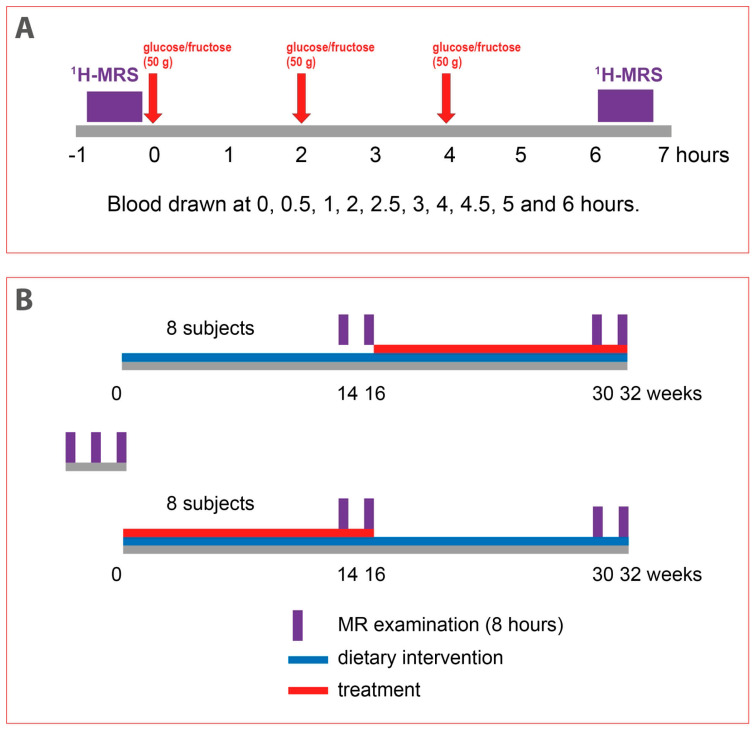
Study design. (**A**). Design of 8 h magnetic resonance (MR) examinations of acute response of hepatic fat to repeated loads of glucose or fructose. (**B**). Design of 32-week intervention study. The order of both dietary interventions was randomized. ^1^H-MRS (proton magnetic resonance spectroscopy).

Sixteen male subjects with obesity were planned to be included in the intervention part of the study. The inclusion criteria were age of 18–70 years, BMI > 30 kg/m^2^, and waist circumference > 102 cm. Exclusion criteria were diabetes mellitus (fasting glucose > 7 mmol/L, antidiabetic treatment), cardiovascular disease and other severe metabolic diseases, glycated hemoglobin (HbA1c) > 48 mmol/mol, triglyceridemia > 4 mmol/L, alcohol consumption > 40 g/day, and fructose intolerance and inability to undergo magnetic resonance (MR) examination. After passing the screening examination, the patients underwent 3 examinations lasting approximately 8 h (Figure 1A). In each of these examinations, liver fat content was measured in the morning after overnight fast. Then, patients received (a) 3 × 50 g of glucose at 2 h intervals, (b) 3 × 50 g of fructose at 2 h intervals, or (c) continued fasting. The liver fat content was then measured again 6 h after the first load of sugar. Blood for biochemical analyses was drawn before and then at 0.5, 1, 2, 2.5, 3, 4, 4.5, 5, and 6 h after first sugar load. Hepatic fat volume (HFV) and the amount of subcutaneous and visceral fat were also determined using MR imaging. The body composition was analyzed by bioimpendance measurement. The order of these three baseline interventions was randomized, and they were carried out at least 2 weeks apart. The subjects with HFC > 4% at first two baseline examinations could then be included in intervention part of the study. The intervention part of the study started immediately after the third baseline examination and consisted of two periods, each lasting 16 weeks—dietary intervention period and treatment period with semaglutide (Figure 1B). Both periods were carried out in a crossover design without washout period. The order of interventions was randomized.

The dietary intervention lasted 16 weeks, but it also continued throughout treatment period. Prior to the intervention, a 7-day dietary record was collected from each subject. After evaluation of this record and evaluation of physical activity and body composition, a certified nutritional therapist provided subjects with personalized recommendations, including an individualized sample menu with an energy balance set to their ideal body weight. The patients were advised to reduce their intake of fat (especially saturated fat) and added sugars and to increase their protein intake with regard to their regular physical activity. The subjects were instructed not to change physical activity throughout the study. Compliance was checked every 4 weeks by nutritional therapist who reviewed the 7-day dietary records and provided feedback to the patients.

During the treatment period, patients were treated with subcutaneous semaglutide (Ozempic^®^, Novo Nordisk A/S, Bagsværd, Denmark) as recommended for treatment of diabetic patients—treatment started at a dose 0.25 mg weekly for 4 weeks, then continued at 0.5 mg weekly for another 4 weeks and finally 1 mg weekly for last 8 weeks. Adherence to treatment was monitored every 4 weeks when patients returned used application pens.

### 2.2. MR Spectroscopy and MR Imaging Examination

Magnetic resonance (MR) protocol consisted of proton MR spectroscopy (^1^H MRS), MR imaging of the liver for quantification of hepatic fat content (HFC) and hepatic volume (carried out before and at the end of each of seven examinations), and MR volumetry of subcutaneous and visceral adipose tissue (once at the baseline and at the end of each intervention periods).

MR examination of the liver was performed in the supine position; all sequences were applied in a held exhalation to maximize the reproducibility of all measurements. Whole-body 3T system Siemens Vida (Siemens Healthineers, Erlangen, Germany) equipped with 30-channel surface and spine coil was used. Imaging part of measurement consisted of the HASTE (half-Fourier acquisition single-shot turbo spin-echo) localizer in three orthogonal planes and VIBE (Volumetric Interpolated Breath-hold Examination) sequences in transversal plane for volumetry of the liver (VIBE e-Dixon sequence: repetition time/echo time (TR/TE) = 3.97/1.29 ms, resolution of 1.2 mm × 1.2 mm × 3 mm, flip angle = 9°, 80 slices, acceleration factor of 2 × 2 caipirinha, and automatic segmentation routine of Siemens). The automatic liver segmentation was checked and manually corrected if necessary.

Hepatic fat fraction (fat signal/fat and water signal) was measured by LiverLab engine consisting of single voxel spectroscopic technique—HISTO (STEAM sequence with following parameters: TR = 3000 ms; 5 spectra during one breath-hold with TE = 12, 24, 36, 48, and 72 ms; voxel size of 40 mm × 30 mm × 25 mm; and bandwidth of 1200 Hz) [12]. Automatic and manual shimming were combined to reach a line halfwidth below 50 Hz. In each subject, the volume of interest (VOI) was always placed in the liver segments V/VIII at the same position, which was carefully controlled during all follow-up examinations by two experienced MR specialists.

To calculate HFV, the following steps were taken: First, hepatic fat fraction from the HISTO protocol was converted to a volume fraction of lipids, known as the HFC, according to Longo [13]. This HFC was then multiplied by the hepatic volume to obtain the HFV.

For MR volumetry of adipose tissue, T2-weighted HASTE sequence in the transversal plane with TR/TE = 1800/91 ms, base resolution = 512, and 3.5 mm slice thickness was applied.

Subcutaneous and visceral adipose tissue was segmented manually in ITK-SNAP from the HASTE sequence from a single slice precisely located in the middle of the 3rd lumbar vertebra. The measurement was carried out once at baseline and at the end of each intervention period.

The entire MR examination took approximately 40 min.

### 2.3. Body Composition

Body composition was measured using Body Composition Analyzer DC-360 (Tanita, Tokyo, Japan).

### 2.4. Characterization of Fatty Acid Profile of Very-Low-Density Lipoprotein (VLDL) Triglycerides

VLDLs were quantitatively isolated from plasma obtained at time 0 of each examination by ultracentrifugation [14]. VLDL lipids were then extracted according to a modified Folch method [15], and triglycerides were separated by thin-layer chromatography. The fatty acid profile of VLDL-triglycerides was then analyzed using gas chromatography [16] with a flame-ionization detector (GC 5890A, Hewlett Packard, Palo Alto, CA, USA). A total of 18 fatty acids were detected and quantified. The proportion of individual fatty acids was then expressed as a percentage of the sum of all fatty acids analyzed.

### 2.5. Biochemical Analyses

Blood was collected into vacutainers with EDTA and immediately chilled on ice. The aliquots of plasma were subsequently stored at −80 °C until analysis. The concentration of TG was determined using enzymatic kits manufactured by Roche Diagnostics (Mannheim, Germany); glucose concentration was determined using a PLIVA Lachema Diagnostika kit (Brno, Czech Republic); cholesterol and levels of alanin aminotransferase (ALT), aspartate aminotransferase (AST), and gamma glutamyltransferase (GGT) were determined using kits from PZ CORMAY S.A., Lomianki, Poland; and non-esterified fatty acid (NEFA) and β-hydroxybutyrate (BHB) concentrations were determined using kits from Wako Chemicals GmbH (Neuss, Germany). Insulin was measured using an IRMA kit (Beckman Coulter, Prague, Czech Republic); the other signaling molecules were measured using ELISA kits (glucagon (Mercodia, Uppsala, Sweden), leptin (BioVendor, Brno, Czech Republic), adiponectin (Millipore, Billerica, MA, USA), FGF-19 (BioVendor, Brno, Czech Republic), FGF-21 (Invitrogen, Carlsbad, CA, USA), and spexin (MyBio Source, San Diego, CA, USA)).

### 2.6. Statistics

The data are presented as mean ± SD. The areas under curve (AUCs) and areas under increment curve (AUICs) for biochemical parameters in plasma were calculated using trapezoid rule. The statistical analyses were carried out using GraphPad Instat 3 (La Jolla, CA, USA). The effect of both interventions was evaluated separately using paired *t*-test or Wilcoxon signed-rank test (based on the normality of the data). When comparing the effect of both interventions, the differences in parameters under comparison were evaluated using the same tests. The differences in AUCs and AUICs in examinations with fructose or glucose were evaluated using ANOVA for repeated measures with Bonferroni correction. The association between variables was evaluated using Pearson’s correlation analysis. A *p* < 0.05 was considered as statistically significant.

## 3. Results

### 3.1. Subjects and Their Baseline Characteristics

A total of 30 males with obesity were screened in the period from June 2020 to February 2022. Seventeen of them passed the screening and were enrolled in the study. One of them withdrew from the study at the first magnetic resonance (MR) examination due to claustrophobia. All of these patients had increased HFC ranging from 6.0 to 34.7% (Table 1) and were therefore eligible for inclusion in the intervention part of the study. Their body mass index (BMI) ranged from 31.1 to 44.1 kg/m^2^, and they were relatively insulin resistant, as documented by HOMA-IR. Except for one subject with an elevated GGT level, they had normal liver enzyme levels (ALT, AST, and GGT).

### 3.2. Semaglutide Treatment Affects Body Weight and Liver Fat

Treatment with semaglutide led to a 6.6 kg reduction in body weight and to corresponding changes in all other adiposity-related parameters (BMI, total body fat as determined by bioimpedance measurement, and the area of visceral and subcutaneous fat measured using MR imaging (MRI)), whereas the dietary intervention alone had no significant effect on these parameters (Table 2). It should be noted that the results were partly affected by the order of the interventions—patients who started with dietary intervention lost 3.2 ± 5.9 kg of weight, but the loss was not statistically significant (*p* = 0.17). After treatment with semaglutide, they lost an additional 6.2 ± 1.9 kg (*p* < 0.001). On the other hand, subjects who started on semaglutide lost 7.1 ± 3.4 kg (*p* < 0.001) after treatment and then gained 3.5 ± 1.8 kg after switching to dietary intervention alone (*p* < 0.001).

Treatment with semaglutide had a pronounced effect on liver fat—hepatic fat content (HFC) decreased by 30% (−3.9 ± 3.6%) and hepatic fat volume (HFV) by 35% (−105 ± 109 mL), whereas dietary intervention had no statistically significant effect on both HFC and HFV (+0.9 ± 2.8%, *p* = 0.706, and +24 ± 69 mL, *p* = 0.353, respectively). Similarly to weight changes, the results were also affected by the order of interventions. Patients who started with dietary intervention experienced a statistically insignificant decrease in both HFC and HFV (−3.6 ± 5.2%, *p* = 0.154, and −100 ± 147 mL, *p* = 0.094, respectively) and then a significant decrease after switching to semaglutide (−4.5 ± 4.4%, *p* = 0.017, and −130 ± 137 mL, *p* = 0.031, respectively). Patients who started on semaglutide had a pronounced decrease in HFC and HFV after treatment (−3.3 ± 2.8%, *p* = 0.011, and −81 ± 73 mL, *p* = 0.016, respectively). Both HFC and HFV then did not change at the end of dietary intervention (+0.9 ± 2.8%, *p* = 0.706, and +24 ± 69 mL, *p* = 0.353, respectively).

Importantly, when we analyzed the relationship between changes in liver fat (evaluated as both HFC and HFV) and changes in body weight and other measures of adiposity, a strong correlation between these parameters was found after dietary intervention but not after treatment with semaglutide (Table 3). Changes in liver fat after semaglutide correlated only with changes in visceral fat.

### 3.3. Semaglutide Treatment Does Not Affect Acute Changes of Liver Fat after Repeated Loads of Glucose and/or Fructose

**Figure 2:**
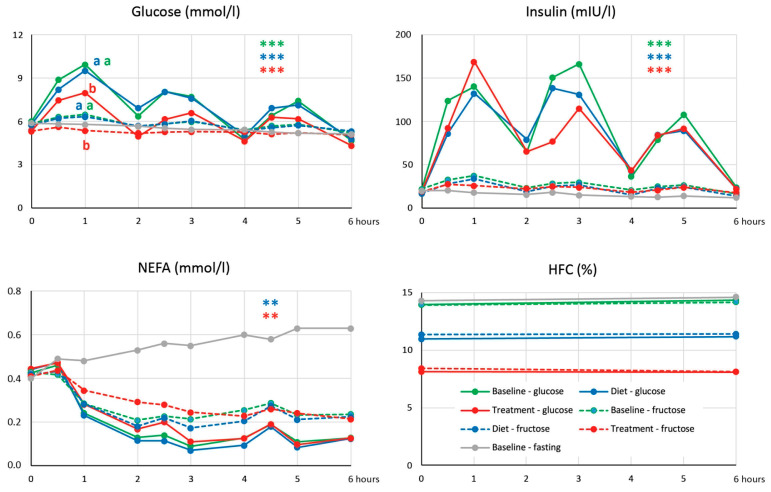
Dynamics of glucose, insulin, and non-esterified fatty acids (NEFA) concentrations and hepatic fat content (HFC) after repeated loads of glucose and fructose. **, *** … *p* < 0.01, *p* < 0.001, respectively, for the difference between response of areas under incremental curves (AUICs) to repeated loads of glucose and fructose at baseline, dietary intervention, and treatment. The same letter (a,b) indicates values of AUIC in experiments with fructose and glucose that do not differ by ANOVA for repeated measures (*p* < 0.05).

No acute HFC changes were induced by repeated administration of both glucose and fructose at baseline, and such a response was unaffected by either dietary intervention or treatment (Figure 2). This observation thus did not support our working hypothesis that a decrease in HFC associated with treatment could be explained by the restoration of HFC decrease in response to repeated loads of glucose.

### 3.4. Semaglutide Treatment Affects Glucoregulation

Treatment with semaglutide led to a significant decrease in plasma glucose, whereas dietary intervention alone had no effect on glycemia (Table 2). On the other hand, the treatment did not have any significant impact on insulinemia, which decreased after dietary intervention. Although the response of HOMA-IR to both treatment and dietary intervention did not differ, the decrease in HOMA-IR was statistically significant only after dietary intervention, not after treatment (*p* = 0.064). However, treatment with semaglutide seems to have a pronounced effect on the insulin sensitivity of tissues, as can be documented by changes in glycemia after the repeated administration of glucose in corresponding examinations (Figure 2). The six-hour area under curve (AUC) of glucose was suppressed after semaglutide treatment compared to baseline and dietary intervention (36.2 ± 3.1 versus 42.6 ± 4.4 and 42.4 ± 4.7 mmol*h/L, respectively, *p* < 0.001) and the same applied to the area under incremental curve (AUIC) of glucose (4.0 ± 3.5 versus 6.5 ± 4.8 and 7.3 ± 3.9 mmol*h/L, respectively, *p* < 0.01). On the other hand, the response of insulinemia in these examinations evaluated as both AUC and AUIC of insulinemia did not differ between these examinations. As expected, treatment with semaglutide and not dietary intervention resulted in a decrease in plasma glucagon.

Both treatment with semaglutide and dietary intervention did not affect fasting cholesterol, NEFA, and β-hydroxybutyrate (BHB) concentrations (Table 2). Semaglutide treatment, and not dietary intervention, led to a decrease in plasma triglycerides (TG) and plasma very-low-density lipoprotein-TG (VLDL-TG).

The treatment with semaglutide resulted in a decrease in plasma leptin concentration and did not affect concentrations of adiponectin, FGF-19, FGF-21, and spexin (Table 2). The dietary intervention had no effect on concentrations of these signaling molecules.

### 3.5. Semaglutide Treatment Affects Fatty Acid Profile of Plasma VLDL-TG

The analysis of the fatty acid profile of plasma VLDL-TG revealed that treatment with semaglutide led to an increase in the proportion of palmitic and palmitoleic acid (16:0 and 16:1n − 7, respectively) and to a decrease in the proportion of linoleic acid (18:2n − 6) (Table 4). The dietary intervention did not affect the fatty acid profile of VLDL-TG. The lipogenic index, defined as a 16:0/18:2n − 6 ratio in VLDL-TG [17], a measure of de novo lipogenesis rate, decreased from 1.36 ± 0.19 to 1.26 ± 0.26 with semaglutide treatment (*p* < 0.05) and was not affected by dietary intervention (1.34 ± 0.29 versus 1.32 ± 0.26).

## 4. Discussion

In our crossover randomized trial, the treatment of non-diabetic patients with obesity with subcutaneous semaglutide (Ozempic^®^) at a dose of 1 mg once weekly for 16 weeks led to a weight loss of 6.6 kg (5.4% of body weight) and a pronounced decrease in liver fat (on average, HFC dropped 30% by 3.9% and HLV 35% by 106 mL). Dietary intervention alone had no significant impact on body weight or liver fat.

These findings are in agreement with previous reports that GLP-1 receptor agonists, including semaglutide, effectively reduce HFC [8,18].

It is generally assumed that a reduction in body weight and an improvement in insulin sensitivity are crucial for the beneficial effects of GLP-1 agonists on liver fat [8]. Interestingly, in our study, we did not observe a significant correlation between changes in HFC and HFV with changes in body weight and other quantitative measures of adiposity in patients treated with semaglutide (Table 3). On the other hand, a significant association between these parameters was found when the subjects used dietary intervention alone. Fatty acids released from adipose tissue are the main source of liver fat [19]; therefore, our data do not seem to support the idea that reduced adipose tissue volume and the resulting reduced supply of fatty acids to the liver is the cause of liver fat loss during semaglutide treatment.

Improved insulin sensitivity may be another factor that may contribute to liver fat reduction after semaglutide. Indeed, in this study, we demonstrated that semaglutide treatment effectively decreases the concentrations of plasma TG and glucose, two principal components of metabolic syndrome, and potently improves glycemic control, as seen by the response of glycemia to repeated glucose loads. However, from the perspective of hepatic fat metabolism, insulin is a potent stimulator of de novo lipogenesis in the liver via activation of the SREBP-1c pathway, and increased insulin sensitivity should lead to the opposite result—hepatic fat accumulation.

Importantly, increased de novo lipogenesis has been shown to be a principal source of hepatic fat in patients with hepatosteatosis [11]. Although we did not measure the rate of de novo lipogenesis directly, we took advantage of the fact that the proportion of palmitoleic acid in VLDL-TG is tightly related to the rate of de novo lipogenesis determined using deuterated water [20]. The finding that the proportion of palmitoleic and also palmitic acids, the two major end products of de novo lipogenesis, is less abundant in VLDL-TG after semaglutide treatment then strongly suggests that a decrease in de novo lipogenesis plays a key role in liver fat loss. The lipogenic index, another parameter related to the de novo lipogenesis rate, also decreased after semaglutide treatment. Such an observation is fully compatible with data in rodents that the expression of genes involved in de novo lipogenesis is indeed suppressed after treatment with GLP-1 receptor agonists [21,22]. Given our data, it is not possible to delineate the exact mechanism involved in such an effect. It can be speculated that the de novo lipogenesis may be directly inhibited by semaglutide via the GLP-1 receptor, but the receptor is not expressed in the liver [23]. Another factor that can affect the rate of de novo lipogenesis is adiponectin, an adipokine secreted from adipose tissue [24]. However, its concentration in plasma was not affected by either dietary intervention or treatment (Table 2), suggesting that adiponectin is not involved in the suppression of de novo lipogenesis by semaglutide. Importantly, GLP-1 receptor agonists also exhibit central effects in reducing appetite and food intake [25], and this also explains the beneficial effect of semaglutide treatment on weight loss [26]. The lower caloric intake (and thus lower intake of all nutrients, including sugars) leads to reduced substrate availability for de novo lipogenesis and may also attenuate signaling through ChREBP and SREBP1c, thereby reducing de novo lipogenesis in spite of improved insulin sensitivity.

Despite a pronounced decrease in HFC after semaglutide treatment, we found no improvement in the acute response of HFC to repeated loads of glucose (Figure 2). However, it must be emphasized that the subjects in whom we observed the decrease in HFC after glucose had a BMI of 26.9 ± 2.7 kg/m^2^ and HFC of 1.9 ± 1.0% [9], which is far from the values reached in our subjects after treatment.

In agreement with previous studies, we observed a decrease in plasma triglycerides after treatment. The mechanism behind such a decrease has not been characterized yet, but we can hypothesize that it can be due to a decrease in liver fat—it has been previously shown that hepatic VLDL_1_-TG production is driven by the amount of liver fat [27] and that VLDL production can be indeed suppressed by treatment with GLP-1 receptor agonist in mice [21].

A reduced intake of food (and thus dietary fat) induced by the administration of semaglutide could also contribute to reduced liver fat content, even if dietary fat is only a minor source of hepatic fat. However, we have no data to support this idea.

It should be noted that due to the choice of exclusion criteria, such as no diabetes and relative normotriglyceridemia, the patients in this study most likely suffered from simple steatosis that had not yet progressed to MASH. This is also evidenced by normal levels of liver enzymes.

Moreover, the semaglutide was used at a dose recommended for the treatment of diabetes, which is lower than that recommended for the treatment of obesity. This may explain the modest impact on weight—our subjects lost only about 5% of weight. In spite of that, they lost approximately a third of their liver fat.

We can assume that the impact of treatment on both body weight and HFC would be more impressive if the dietary intervention was combined with increased physical activity. It will likely improve the results of the control intervention period without drugs. However, we asked the subjects not to change their physical activity and lifestyle, as the induced metabolic changes can be quite complex and may vary between individuals.

A certain limitation of our study is the fact that we did not use a washout period in the crossover design. We decided to skip the washout because we would have to include two more examinations at the beginning of the second intervention period. We believe that the results of a dietary intervention could be significantly improved if washout is included. Another limitation of our study is the fact that it was conducted only in males. However, when enrolling female subjects, it would be necessary to take into account the cycle, hormonal contraception, and menopause, which would disproportionately increase the size of the study group.

To the best of our knowledge, our study used, for the first time, the MR and analysis of fatty acid profile in VLDL-TG to analyze in more detail the mechanisms involved in the effect of semaglutide on hepatic fat in obese subjects with MASLD and introduced evidence that the suppression of de novo lipogenesis plays a role in decreasing liver fat in humans treated with semaglutide.

There is currently an intense search for drugs that could improve inflammation and fibrosis in patients with advanced stages of MASLD. Semaglutide and other GLP-1 receptor agonists appear to be promising candidates for such treatment. However, our data suggest that treating obese individuals who have not yet progressed to the late stages of the disease with semaglutide may be even more important. Reducing liver fat in these patients could prevent associated lipotoxicity and thus prevent the progression of MASLD.

In conclusion, our data suggest that successful treatment of obesity with semaglutide also leads to a pronounced reduction of liver fat, even in subjects in the early stages of MASLD development. The suppression of de novo lipogenesis seems to play an important role in such an effect of semaglutide. However, due to a pleiotropic mode of action of semaglutide and GLP-1 receptor agonists in general on the energy homeostasis and metabolic processes, it is possible that other as yet unidentified mechanisms may have contributed.

## Figures and Tables

**Table 1:** Baseline characteristics of subjects. Data are mean ± SD. BMI (body mass index), HOMA-IR (homeostatic model assessment of insulin resistance), ALT (alanine aminotransferase), AST (aspartate aminotransferase), and GGT (gamma-glutamyl transferase).

n	16
Age (years)	47.0 ± 12.4
Weight (kg)	120.3 ± 12.0
BMI (kg/m^2^)	36.2 ± 3.8
Waist circumference (cm)	120 ± 10
Total body fat (kg)	39.4 ± 7.9
Total body fat (%)	32.4 ± 3.7
Glucose (mmol/L)	5.92 ± 0.47
Insulin (mIU/L)	21.1 ± 6.9
HOMA-IR	5.24 ± 1.64
Triglyceride (mmol/L)	1.36 ± 0.29
Cholesterol (mmol/L)	4.51 ± 0.68
ALT (µkat/L)	0.60 ± 0.15
AST (µkat/L)	0.48 ± 0.12
GGT (µkat/L)	0.75 ± 0.83
Hepatic fat content (%)	13.3 ± 8.5

**Table 2:** Effects of dietary intervention and treatment with semaglutide on selected anthropometric and metabolic parameters. Data are mean ± SD. HFC: hepatic fat content, HFV (hepatic fat volume), TG (triglycerides), VLDL-TG (very-low-density lipoprotein-triglycerides), NEFA (non-esterified fatty acids), BHB (β-hydroxybutyrate), and FGF (fibroblast growth factor). *, **, *** … *p* < 0.05, *p* < 0.01, *p* < 0.001, respectively, start vs. end of the interventions.

	Dietary Intervention		Treatment		Diet vs. Treatment
	Start	End	Start	End	*p*
Adiposity					
Weight (kg)	116.8 ± 13.0	117.0 ± 11.6	118.7 ± 12.3	112.1 ± 12.4 ***	0.0008
BMI (kg/m^2^)	35.1 ± 4.0	35.2 ± 3.5	35.7 ± 3.9	33.7 ± 3.8 ***	0.0008
Total body fat (kg)	36.7 ± 8.4	37.0 ± 7.6	38.5 ± 8.3	34.0 ± 8.1 ***	0.0001
Total body fat (%)	31.1 ± 4.1	31.4 ± 3.8	32.1 ± 4.0	30.1 ± 4.0 ***	0.0002
Subcutan. fat (cm^2^)	372 ± 124	380 ± 130	391 ± 140	349 ± 131 ***	0.0042
Visceral fat (cm^2^)	318 ± 72	318 ± 70	322 ± 70	293 ± 63 **	0.0052
Liver fat					
HFC (%)	12.35 ± 8.77	11.02 ± 8.06	12.21 ± 7.56	8.33 ± 4.98 ***	0.0250
HFV (mL)	276 ± 230	238 ± 198	266 ± 186	161 ± 99 ***	0.0507
Biochemistry					
HOMA-IR	5.26 ± 1.73	4.43 ± 1.58 *	5.43 ± 2.19	4.55 ± 1.77	0.9439
Insulin (mIU/L)	20.6 ± 6.2	17.1 ± 5.5 *	20.5 ± 7.5	18.9 ± 6.6	0.4331
Glucagon (pmol/L)	7.16 ± 3.06	7.80 ± 3.50	8.35 ± 3.08	5.98 ± 2.60 ***	0.0007
Glucose (mmol/L)	5.71 ± 0.53	5.80 ± 0.46	5.91 ± 0.46	5.35 ± 0.34	<0.0001
TG (mmol/L)	1.25 ± 0.31	1.36 ± 0.47	1.40 ± 0.44	1.14 ± 0.32 **	0.0166
VLDL-TG (mmol/L)	0.83 ± 0.27	0.99 ± 0.43	0.99 ± 0.39	0.76 ± 0.32 **	0.0115
NEFA (mM)	0.45 ± 0.12	0.43 ± 0.13	0.41 ± 0.14	0.43 ± 0.09	0.1297
ΒHB (µmol/L)	45.1 ± 22.3	37.8 ± 13.5	41.1 ± 21.0	46.8 ± 22.0	0.3484
Cholesterol (mmol/L)	4.63 ± 0.74	4.38 ± 0.72	4.56 ± 0.68	4.56 ± 0.90	0.2070
Leptin (µg/L)	58.3 ± 27.6	58.6 ± 25.7	65.1 ± 31.0	49.1 ± 27.0 ***	0.0125
Adiponectin (µg/L)	6046 ± 2135	6127 ± 1883	5876 ± 1940	6102 ± 2179	0.7236
FGF-19 # (ng/L)	196 ± 168	189 ± 149	164 ± 91	206 ± 165	0.3390
FGF-21 ## (ng/L)	209 ± 135	192 ± 109	193 ± 109	184 ± 119	0.7936
Spexin (ng/L)	130 ± 77	131 ± 70	131 ± 87	123 ± 78	0.3303
ALT (µkat/mmol)	0.64 ± 0.17	0.62 ± 0.27	0.62 ± 0.24	0.54 ± 0.28	0.4164
AST (µkat/mmol)	0.46 ± 0.13	0.45 ± 0.11	0.46 ± 0.11	0.43 ± 0.12	0.6519
GGT (µkat/mmol)	0.66 ± 0.82	0.66 ± 0.65	0.70 ± 0.67	0.62 ± 0.79 *	0.1167

#, ## … n = 11, n = 13, respectively.

**Table 3:** Pearson correlation coefficients between change in liver fat measured as hepatic fat content (Δ HFC) or hepatic fat volume (Δ HFV) and change in selected anthropometric and metabolic parameters after dietary intervention and after treatment with semaglutide. *, **, *** … *p* < 0.05, *p* < 0.01, *p* < 0.001, respectively.

	Dietary Intervention		Treatment	
	Δ HFC (%)	Δ HFV (mL)	Δ HFC (%)	Δ HFV (mL)
Δ HFC (%)	1.000 ***	0.974 ***	1.000 ***	0.945 ***
Δ HFV (mL)	0.974 ***	1.000 ***	0.945 ***	1.000 ***
Δ weight (kg)	0.888 ***	0.836 ***	0.284	0.230
Δ BMI (kg/m^2^)	0.903 ***	0.849 ***	0.338	0.274
Δ total body fat (kg)	0.882 ***	0.840 ***	0.021	−0.078
Δ total body fat (%)	0.834 ***	0.804 ***	0.011	−0.174
Δ subcutaneous fat (cm^2^)	0.551 *	0.541 *	0.441	0.303
Δ visceral fat (cm^2^)	0.781 **	0.683 **	0.720 **	0.672 **
Δ HOMA-IR	0.226	0.209	−0.319	−0.182
Δ insulin	0.201	0.179	−0.401	−0.257
Δ glucagon	0.330	0.314	−0.059	−0.077
Δ glucose	0.189	0.195	0.219	0.288
Δ TG	0.329	0.351	−0.346	−0.264
Δ VLDL-TG	0.312	0.337	−0.507 *	−0.393

**Table 4:** Effects of dietary intervention and treatment with semaglutide on proportional representation of individual fatty acids in VLDL-triglycerides. * … *p* < 0.05 end vs. start of intervention.

	Dietary Intervention		Treatment		Diet vs. Treatment
Fatty Acid	Start	End	Start	End	*p*
14:0	0.52 ± 0.13	0.49 ± 0.12	0.47 ± 0.12	0.50 ± 13	0.1548
16:0	23.34 ± 1.99	23.05 ± 1.97	23.44 ± 1.74	22.60 ± 1.59 *	0.3149
18:0	3.59 ± 0.81	3.39 ± 0.83	3.44 ± 0.87	3.34 ± 0.81	0.7889
16:1n − 7	2.82 ± 0.92	3.02 ± 0.81	2.85 ± 0.80	2.50 ± 0.51 *	0.0229
18:1n − 9	42.64 ± 3.12	42.75 ± 2.29	43.09 ± 3.00	43.10 ± 3.30	0.9075
18:1n − 7	3.02 ± 0.58	3.37 ± 0.75	3.31 ± 0.55	3.17 ± 0.62	0.0833
18:2n − 6	17.93 ± 2.87	17.82 ± 2.57	17.43 ± 2.06	18.35 ± 2.96 *	0.2510
18:3n − 6	0.32 ± 0.14	0.33 ± 0.11	0.33 ± 0.13	0.32 ± 0.10	0.5981
20:2n − 6	0.39 ± 0.06	0.41 ± 0.07	0.40 ± 0.07	0.40 ± 0.09	0.4159
20:3n − 6	0.40 ± 0.09	0.42 ± 0.09	0.40 ± 0.09	0.41 ± 0.10	0.7185
20:4n − 6	1.39 ± 0.35	1.37 ± 0.32	1.47 ± 0.39	1.42 ± 0.34	0.7248
22:4n − 6	0.23 ± 0.05	0.24 ± 0.06	0.25 ± 0.08	0.23 ± 0.05	0.1386
18:3n − 3	1.42 ± 0.77	1.41 ± 0.73	1.32 ± 0.69	1.48 ± 0.67	0.0833
18:4n − 3	0.31 ± 0.11	0.33 ± 0.08	0.33 ± 0.08	0.31 ± 0.09	0.3261
20:4n − 3	0.05 ± 0.01	0.05 ± 0.02	0.05 ± 0.02	0.05 ± 0.02	0.6206
20:5n − 3	0.27 ± 0.17	0.27 ± 0.13	0.26 ± 0.15	0.28 ± 0.19	0.8443
22:5n − 3	0.15 ± 0.05	0.16 ± 0.05	0.16 ± 0.05	0.16 ± 0.05	0.7511
22:6n − 3	0.51 ± 0.16	0.52 ± 0.14	0.48 ± 0.15	0.54 ± 0.19	0.3750

## Data Availability

The data presented in this study are available upon request from the corresponding author.
